# Efficient Photocatalysts Made by Uniform Decoration of Cu_2_O Nanoparticles on Si Nanowire Arrays with Low Visible Reflectivity

**DOI:** 10.1186/s11671-018-2735-7

**Published:** 2018-10-04

**Authors:** Chien-Hsin Tang, Po-Hsuan Hsiao, Chia-Yun Chen

**Affiliations:** 1 Department of Materials Science and Engineering, National Cheng Kung University, Tainan, 70101 Taiwan; 2 Hierarchical Green-Energy Materials (Hi-GEM) Research Center, National Cheng Kung University, Tainan, 70101 Taiwan

**Keywords:** Photocatalysts, Silicon nanowires, Electroless deposition, Copper oxide

## Abstract

Highly uniformed decorations of Cu_2_O nanoparticles on the sidewalls of silicon nanowires (SiNWs) with high aspect ratio were prepared through a two-step electroless deposition at room temperature. Morphology evolutions and photocatalytic performance of SiNWs decorated with aggregated and dispersed Cu_2_O nanoparticles were unveiled, and the correlated photodegradation kinetics was identified. In comparison with the conventional direct loadings where the aggregated Cu_2_O/SiNW structures were created, the uniform incorporation of Cu_2_O with SiNWs exhibited more than three and nine times of improved photodegradation efficiency than the aggregated-Cu_2_O/SiNWs and sole SiNWs, respectively.

**Electronic supplementary material:**

The online version of this article (10.1186/s11671-018-2735-7) contains supplementary material, which is available to authorized users.

## Background

Cu_2_O nanostructures, with direct band gap energy of 2.0–2.2 eV, have been emerged as the efficient photocatalytic materials that could decompose the organic pollutants directly through the activation of visible light [1–4]. The benefited effects also correlated with their environmental acceptability, low toxicity, and sustainable availability, which could be potential for many practical applications including hydrogen production, solar cells, and chemical sensing [5–7]. Nevertheless, these features were limited by the usage instability and unrepeatability due to the intended aggregation or significant morphological changes of nanostructures upon operation within an aqueous environment. In this regard, the supporters that enabled to disperse the photoactive Cu_2_O nanostructures in a sable manner were considered to be highly desirable, which could maintain the long-term capability of photodegradation process of organic pollutants and represented the reproducible, efficient, and reliable platform for the practical requirement of photocatalytic operation.

Silicon nanowires (SiNWs), with good surface hydrophilicity, mechanical robustness, and chemical stability could be the potential supporting materials for strengthening the photocatalytic activity of Cu_2_O nanostructures, rendering a promising design of visible-responsive photocatalysts [8–12]. Moreover, SiNWs further possessed highly visible-absorption capability due to confining the incident lights through the effects of multiple scattering [11]. However, the difficulties of processing lied in the deposition non-uniformity of Cu_2_O nanoparticles on the sidewalls of SiNWs using the inexpensive solution-based processing because of the nature of high aspect ratio in SiNWs. Therefore, in this study, such limitations were overcome by adapting the two-step electroless deposition in order to achieving uniform incorporation of Cu-oxide nanoparticles with the SiNW arrays. In addition, the controlled formation of heterostructural n-type Cu_2_O/p-type Si could be particularly potential to act as efficient photocatalysts [13] because the photo-excited electrons and holes might be separated to initiate photodegradation reaction of dye removal prior to rapid carrier recombination [14, 15]. Based on such designs, investigations of surface morphologies, chemical compositions, and crystallographic analysis were performed to characterize the synthesized Cu_2_O/Si nanostructures.

Next, light-reflection and photoluminescent properties of Cu_2_O/SiNW arrays were measured to identify the light properties and examine the influences of adding Cu_2_O nanoparticles on the decreased recombination of photogenerated carriers. In addition, photocurrent measurements were further performed to clarify the carrier separation of Cu_2_O/SiNW heterostructures under light illuminations. Finally, the detailed photocatalytic evaluations were performed, which clarified the efficient photocatalytic reactivity of degrading organic dyes and explained the involving photodegradation mechanism of such nanostructured photocatalysts.

## Methods/Experimental

### Materials

The utilized Si substrate was p-type Si (100) made with Czochralski process. Silver nitrate (99.85%, Acros Organics, Geel, Belgium), hydrofluoric acid (48%, Fisher Scientific UK, Loughborough, UK), and nitric acid (65%, AppliChem PanReac, Germany) were used for the fabrication of Si nanowire arrays. CuSO_4_ (98+%, Acros Organic, Geel, Belgium) and hydrofluoric acid were used in the synthesis of Cu_2_O nanoparticles. Methylene blue (pure, Acros Organics, Geel, Belgium) was utilized for photodegradation test.

### Fabrication of Si Nanowire Arrays

SiNW arrays were prepared by dipping the as-cleaned p-type Si (100) substrates in the mixed solutions (20 ml) containing 0.02 M of AgNO_3_ and 4.8 M of HF under a gentle magnetic stir at room temperature. Subsequently, the samples were rinsed with deionized (DI) water and then immersed in the concentrated nitric acid (63%) for 15 min in order to completely remove the residual Ag particles. Finally, the as-prepared SiNWs were rinsed with DI water and preserved in the vacuum chamber.

### Synthesis of Cu_2_O Nanoparticles

Electroless deposition of Cu was made by two distinct methods. In method 1, the as-etched SiNWs were directly immersed in the aqueous solutions (20 ml) with 0.047 g (0.015 M) of CuSO_4_ powders and 4.5 M of HF for 3 min. This facilitated the reduction of Cu^2+^ ions preferentially at the primarily created Cu aggregates and therefore limited the well incorporation of Cu_2_O nanoparticles on SiNW arrays. The as-prepared samples were described as aggregated-Cu_2_O/SiNWs (A-Cu_2_O/SiNWs). In method 2, the as-prepared Si nanowires was dipped in 0.015 M of CuSO_4_ solutions (20 ml) for 15 min and followed by gently introducing HF (4.5 M) for initiating the reduction of Cu^2+^ ions. Through the separation of introducing Cu^2+^ ions and HF etchants in the sequential processes, each Cu^2+^/Si interface were activated for the electroless reduction of Cu^2+^ ions, which essentially led to the formation of well-dispersed Cu nanoparticles directly on SiNW arrays. The formed nanostructures prepared with method 2 were described as dispersed-Cu_2_O/SiNWs (D-Cu_2_O/SiNWs). After conducting the electroless deposition, all the fabricated samples were rinsed in DI water and then subjected to the oven at 90 °C for thermal oxidation (30 min).

### Characterizations

Morphology and chemical compositions of as-prepared photocatalysts were characterized with field emission scanning electron microscopy (FESEM; Hitachi JSM-6390) and energy-dispersive X-ray (EDX) spectrometer (Oxford INCA 350), respectively. Prior to SEM investigation, the samples were deposited with a thin layer of Au to improve imaging resolution. Transmission electron microscope (TEM; JEM-2100F) was further performed to characterize the surface morphologies of samples. The samples were carefully scratched from substrates and dispersed in ethanol using an ultrasonicator, and then the dispersed solutions were dipped on the TEM grids. Crystallographic characterizations were performed with Rigaku Multiflex X-ray diffractometer using the Cu-K radiation. Light reflection spectra were measured with a UV-Vis-NIR spectrophotometer (Varian, Cary 5000, Australia). Photocatalytic experiments of various hybrid photocatalysts were conducted with a PanChum multilamp photoreactor (PR-2000) under the illuminations of light source with center wavelength of 580 nm. In each test, 0.2 mM of methylene blue (MB) was utilized as the tested targets. Prior to the light irradiations, the samples were placed in the dark for 40 min in order to establish the adsorption equilibrium, as presented in the Additional File 1. In photodegradation tests, at each time interval, 0.1 ml of suspension was withdrawn and then diluted with 5 ml distilled water. The concentrations of MB dyes were evaluated with UV/visible spectrophotometer (Shimadzu UV-2401 PC).

## Results and Discussion

**Fig. 1:**
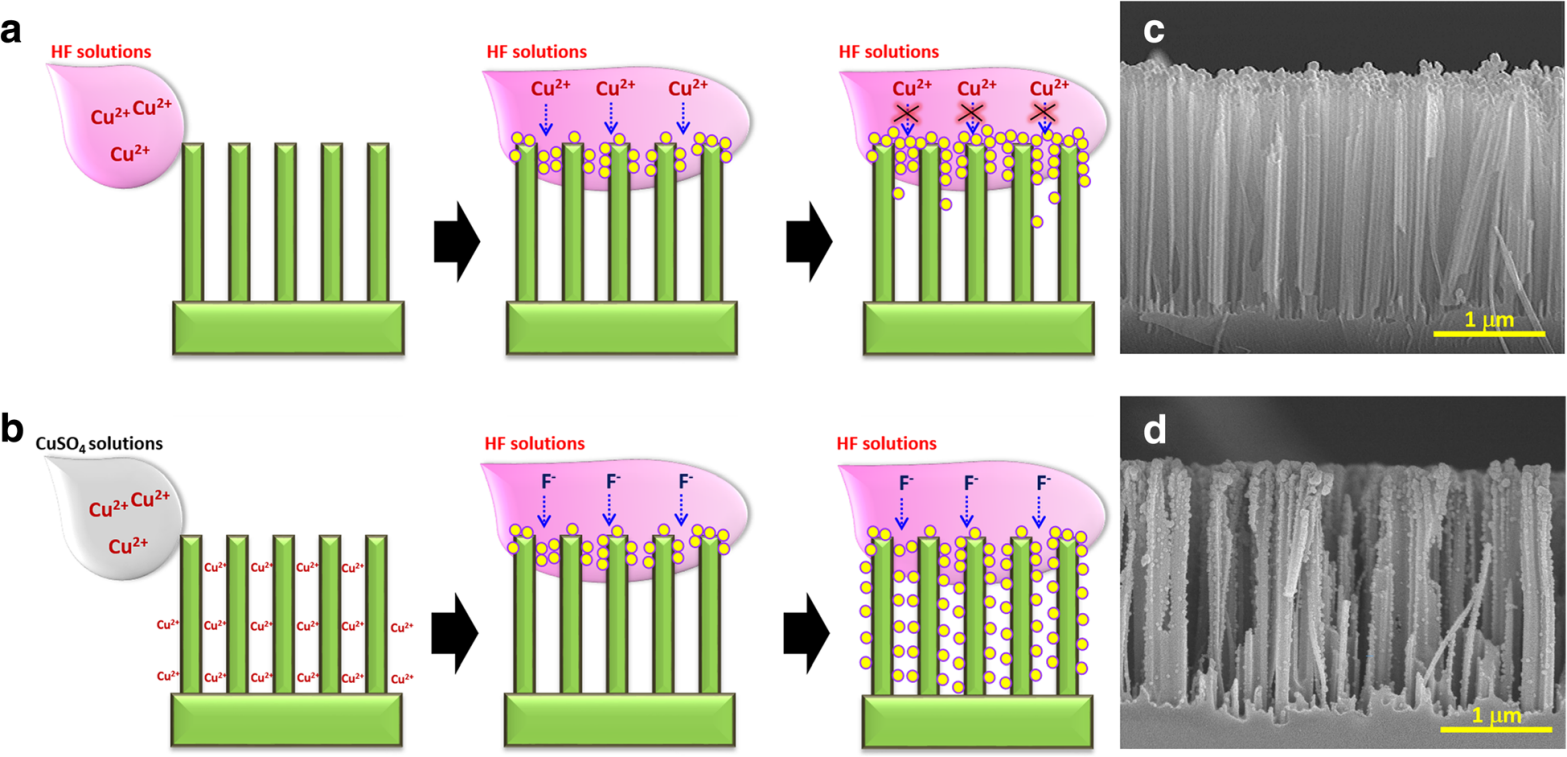
Schematic illustrations for the formation of **a** aggregated Cu_2_O and **b** dispersed Cu_2_O decorated Si nanowires. **c**, **d** The SEM images of aggregated Cu_2_O/SiNW arrays (A-Cu_2_O/SiNWs) and dispersed Cu_2_O/SiNW arrays (D-Cu_2_O/SiNWs), respectively

Figure 1 illustrated two different electroless deposition of Cu on SiNWs, which allowed the formation of distinct deposition morphologies. By directly dipping the SiNWs samples which were fabricated with silver-assisted chemical etching [16–23] into the mixed Cu^2+^/HF solutions, the immediate reduction of Cu^2+^ ions directly on the exposed nanowire tips were taken place, as shown below,

1 $$ {\mathrm{Cu}}^{2+}+2{\mathrm{e}}^{\hbox{-}}\kern0.5em \to \kern0.5em \mathrm{Cu}\kern2.75em {\mathrm{E}}^0=+0.34\ \mathrm{V}\kern1.00em $$

2 $$ {\mathrm{Si}}_{\left(\mathrm{s}\right)}+2{\mathrm{H}}_2\mathrm{O}\kern0.5em \to \kern0.5em {\mathrm{Si}\mathrm{O}}_{2\left(\mathrm{s}\right)}+4{\mathrm{H}}^{+}+4{\mathrm{e}}^{\hbox{-} \kern5.5em }{\mathrm{E}}^0=\hbox{-} 1.24\ \mathrm{V} $$

3 $$ {\mathrm{SiO}}_2+\mathrm{HF}\kern0.5em \to \kern0.5em {\mathrm{H}}_2{\mathrm{SiF}}_6+2{\mathrm{H}}_2\mathrm{O} $$

Thus, it resulted in the Cu aggregations on the top surfaces of SiNWs. The succeeding reduction of Cu^2+^ ions was preferentially occurred nearby these primarily formed Cu aggregates, where the effective transfer of holes from newly arrived Cu^2+^ ions into Si was facilitated, as presented in Fig. 1a. Meanwhile, the diffusion of Cu^2+^/HF reactants turned to hardly reach the bottom sides of SiNWs owing to the involved steric hindrance of the existed Cu nanoparticles. These combined effects might limit the possible formation of well decoration of Cu_2_O nanoparticles on SiNW arrays after experiencing the heat treatment, which therefore degraded their photocatalytic activity.

Figure 1b presented the possible electroless pathway that significantly reduced the aggregated formation of Cu nanoparticles which were locally confined on the nanowire tips. This was accomplished by separating the introductions of Cu^2+^ ions and HF etchants in the sequential processes. Accordingly, the incorporation of Cu^2+^ ions with SiNWs facilitated the uniform reduction of Cu^2+^ ions initiated by adding HF solutions, where each Cu^2+^/Si interface could be activated for the electroless deposition. Evidently, the distinct morphologies of Cu_2_O nanoparticles formed on SiNW arrays were presented in Fig. 1c, d, respectively. In comparison with the aggregated Cu_2_O nanoparticles dominantly at the nanowire tips (Fig. 1c), the incorporations of Cu_2_O nanoparticles with SiNW arrays prepared with two-step electroless deposition appeared to be highly uniformed throughout the sidewalls of nanowires, as shown in Fig. 1d. These features could be clearly observed from the high-magnification SEM image, as presented in the Additional File 1.

**Fig. 2:**
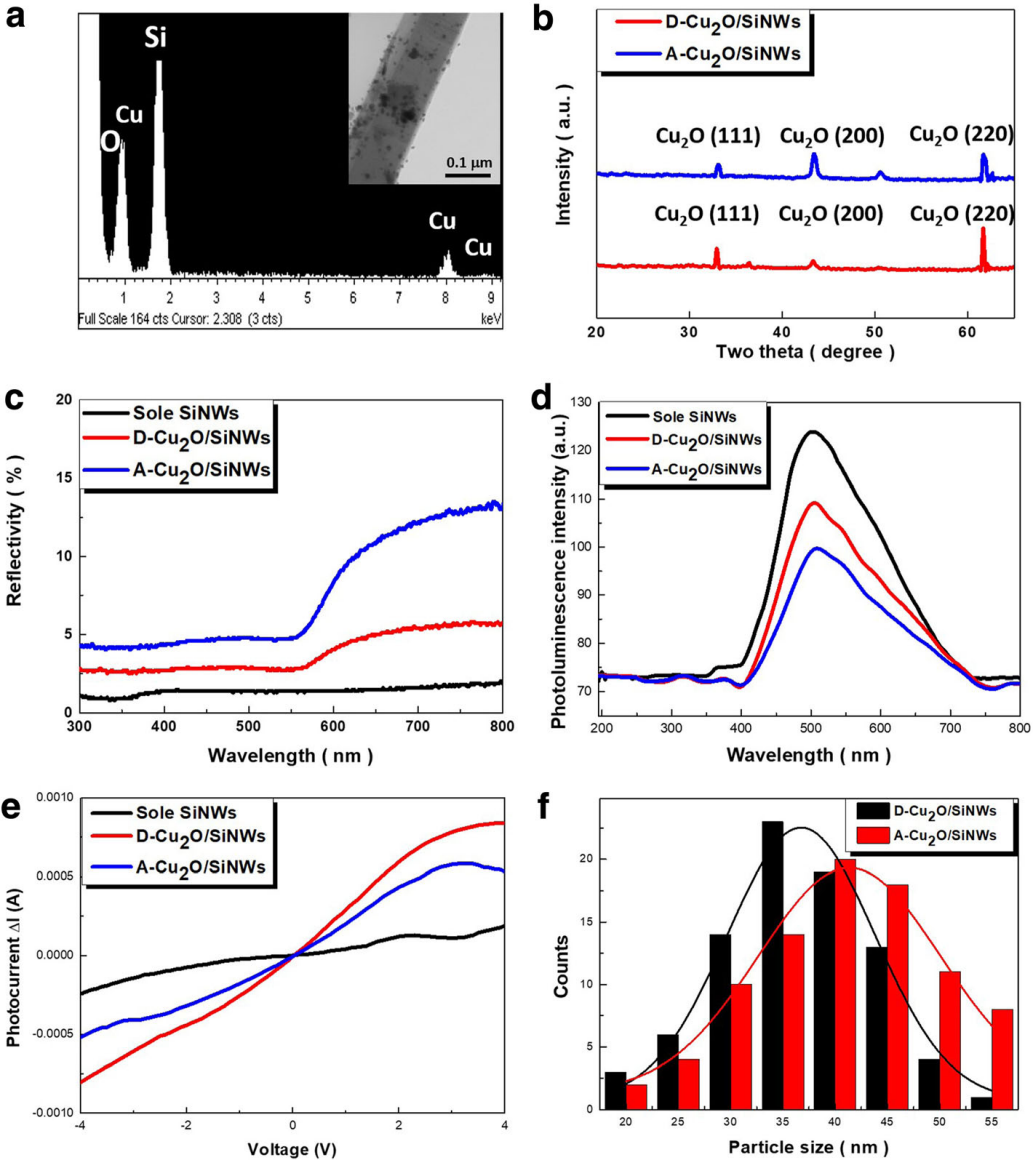
**a** EDS spectrum of dispersed Cu_2_O/SiNW arrays. The insert figure was the corresponding TEM image. **b** XRD analysis, **c** light reflectivity and **d** photoluminescent results, **e** photocurrent enhancement (*I*_photocurrent_ − *I*_dark current_) and **f** size distributions of the samples

Micro-composition characterizations were further examined using energy-dispersive X-ray (EDS) analysis, showing the three characteristic elemental peaks, O, Cu, and Si without the observations of other components, as demonstrated in the Fig. 2a. Moreover, a representative TEM investigation of Cu_2_O-decorated Si nanowires further clarified the robust formation of these hybrid nanostructures with sound spatial distributions of decorated nanoparticles, as shown in the insert of Fig. 2a. To characterize the crystallographic of formed Cu_2_O nanoparticles, XRD analysis were performed, as shown in Fig. 2b. The results clearly identified the characteristic Cu_2_O diffraction patterns with correlated plane indexes of (111), (200), and (220) appearing on both aggregated (Fig. 1c) and dispersed (Fig. 1d) Cu_2_O/SiNW arrays, respectively. The peak at 51° in the XRD patterns was originated from (200) planes of crystalline Cu due to the inner side of Cu_2_O that were incapable of being completely oxidized via the thermal treatment. In addition, the resulting optical reflectivity was measured, indicating that the distinct light reflection properties of aggregated and dispersed Cu_2_O/SiNW arrays, as presented in Fig. 2c. The very low light reflectivity of dispersed Cu_2_O/SiNW arrays whose average reflectivity was 3.8%, which was only slightly higher that of sole SiNW arrays (average reflectivity = 1.4%).

Nevertheless, the aggregated Cu_2_O/SiNW arrays possessed the high reflectivity covering the whole measured spectral regions (average reflectivity = 7.7%) due to the strong reflection of incoming lights directly from the Cu_2_O aggregates on SiNW tops, which could significantly degrade the effective lights/photocatalysts interaction. In addition, photoluminescent (PL) analysis was performed. It was found that all the samples demonstrated the PL peak centered at 522 nm, whereas the corresponding PL intensities of both D-Cu_2_O/SiNWs and A-Cu_2_O/SiNWs were much lower than that of sole SiNWs, as shown in Fig. 2d. These features implied that the recombination of photogenerated carriers was greatly reduced due to the introduction of Cu_2_O/SiNW heterostructures. On the other hand, compared with D-Cu_2_O/SiNWs, the slightly lower PL intensity was observed from A-Cu_2_O/SiNWs, which could be attributed to the strong reflection of incoming lights occurred at top Cu_2_O aggregates, thus decreasing the possibility of light emission. The possible interaction pathways between incoming lights and samples could be found in the Additional File 1. Moreover, the measurements of photocurrents were performed to clarify the carrier separation of Cu_2_O/SiNW heterostructures under light illuminations, as presented in Fig. 2e. It was found that the photocurrent enhancement, evaluated by *I*_photocurrent_ − *I*_dark current_, was 0.216 mA (sole SiNWs), 0.527 mA (A-Cu_2_O/SiNWs), and 0.823 mA (D-Cu_2_O/SiNWs) at bias of 4 V. These results clearly supported our findings from light reflectivity and PL measurements, where the effective separation of photoexcited carriers occurred at D-Cu_2_O/SiNWs that contributed to the improved photocurrent enhancement.

**Fig. 3:**
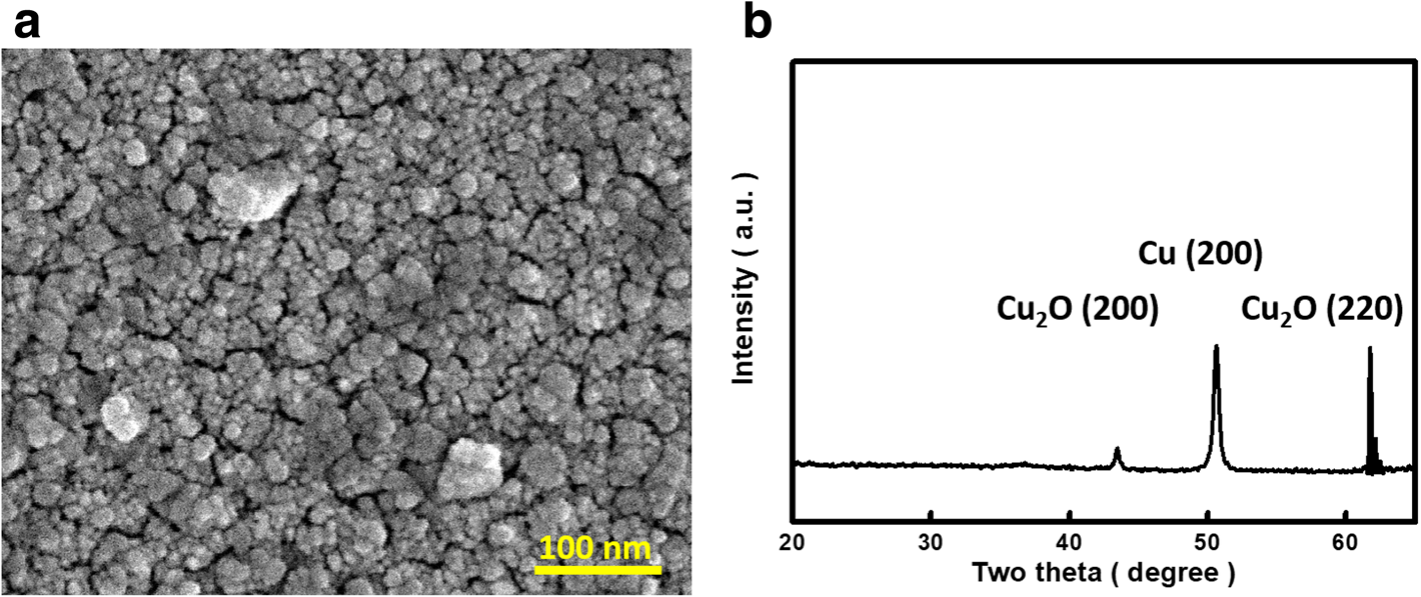
**a** Top-view SEM image and **b** XRD pattern of planar Si substrates coated with Cu_2_O nanoparticles

Particle sizes of formed Cu_2_O were further examined, as demonstrated in Fig. 2f. It was found that the size distributions of Cu_2_O nanoparticles by two different methods described in Fig. 1a, b were fairly close to each other, where the average dimensions evaluated with Gaussian fittings from aggregated and dispersed Cu_2_O/SiNW arrays were 41.5 nm and 36.4 nm, respectively. This might explain the similar nucleation mechanism of Cu nanoparticles from these two deposition methods. In addition, Fig. 3 demonstrated the morphologies and XRD results of Cu_2_O-loaded planar Si. In comparison with abundant surfaces provided by high aspect ratio SiNWs which facilitated the creation of numerous hetero-nucleation sites of Cu_2_O seeds, the comparably large sizes of Cu_2_O nanoparticles with average dimensions of 64.2 nm could be formed on planar Si substrates, as evidenced in Fig. 3a. Moreover, from the XRD patterns one could observe the obvious metallic Cu (200) diffraction peaks, which identified the incomplete transition of copper oxidation via a heat treatment because of the existence of highly dense features of generated seeds through electroless Cu deposition, as presented in Fig. 3b. This again clarified the influences of incorporating Si nanostructures for assisting the generation of functional Cu_2_O nanoparticles with sound spatial distributions.

**Fig. 4:**
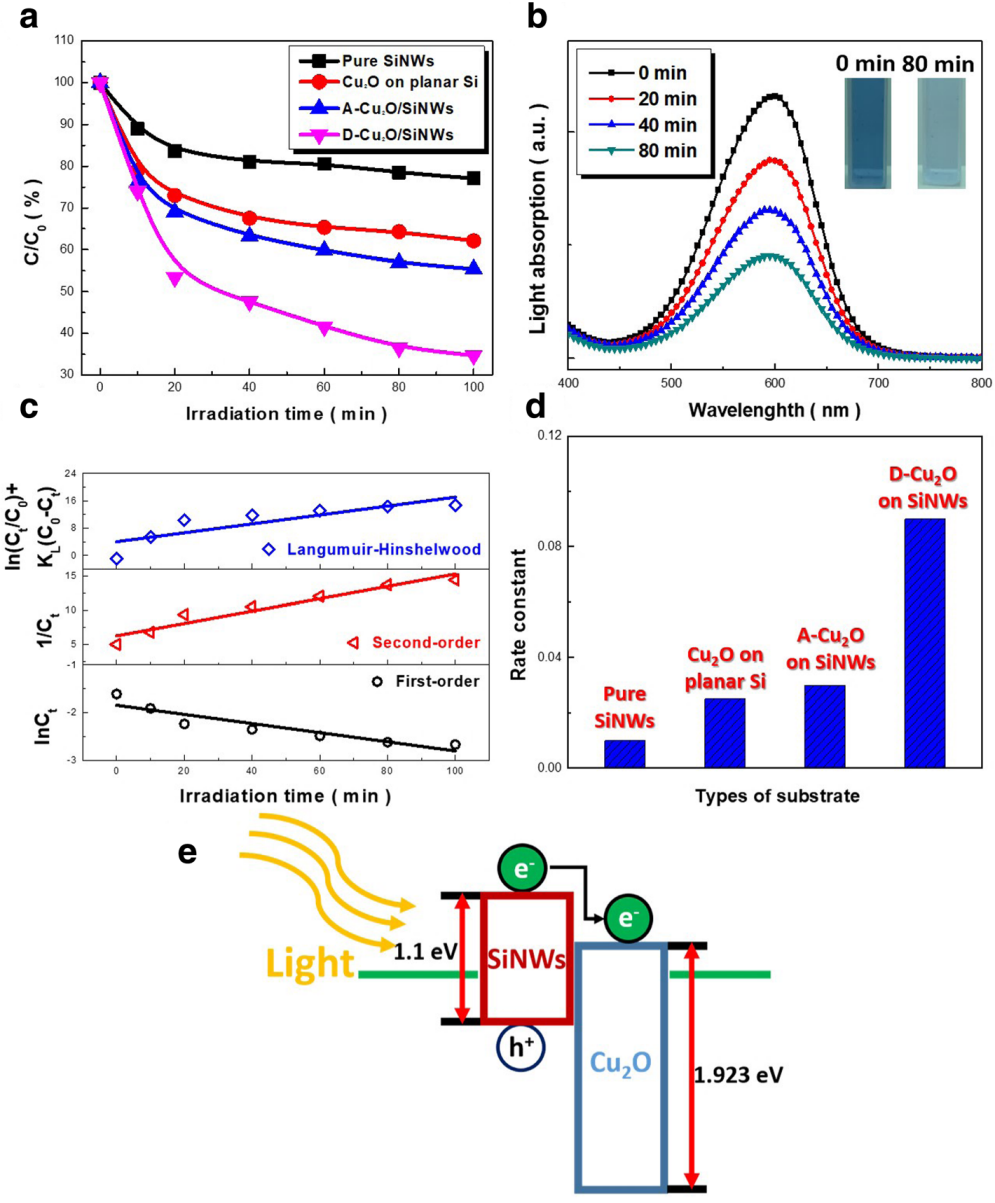
**a** Photocatalytic tests of four various photocatalysts. **b** Changes of absorption spectra and **c** the correlated kinetic modeling in the presence of D-Cu_2_O/SiNW arrays at various durations of light illuminations. The insert figure in **b** presented the color change of dye solutions from 0 to 80 min. **d** Comparisons of rate constants of photodegradation. **e** Band diagram of Cu_2_O/SiNW heterostructures

Evaluations of photocatalytic activity of Cu_2_O decorated SiNW arrays with distinct deposition morphologies were demonstrated in Fig. 4a. In the control experiments, similar measurements were also performed in the presence of unloaded SiNW arrays and Cu_2_O-loaded planar Si, respectively. It could be clearly observed that the D-Cu_2_O/SiNWs photocatalysts presented the superior photoactive degradation of MB dyes, and the significant decrease of absorption peak of MB dyes in the presence of D-Cu_2_O/SiNWs under light illuminations could also be found in Fig. 4b. From the comparisons presented in Fig. 4a, the remaining MB dyes after 100-min reaction are 34.7% in D-Cu_2_O/SiNWs, 55.4% in A-Cu_2_O/SiNWs, 62.1% in Cu_2_O-loaded planar Si, and 77.1% in pure SiNWs, respectively. Under light illuminations, the photo-activated SiNWs could generate electron and hole pairs. The charge recombination would then dominate the photochemical reactions, and therefore [24], the resulting photodegradation efficiency was greatly limited in the presence of pure SiNW array as photocatalysts. With the decoration of Cu_2_O nanoparticles on SiNWs, the electron-hole recombination was effectively retarded through scavenging the photogenerated electrons [25, 26]. This conclusion could be supported by the improved photodegradation rates of three Cu_2_O-incorporated Si, as shown in Fig. 4a. Moreover, the aggregated features of Cu_2_O nanoparticles on SiNW tips would inhibit the effective absorption of light, thus suppressing the activity of photocatalysts for dye degradation, as found in the photocatalytic test of A-Cu_2_O/SiNWs. In addition, from the scavenger analysis, we confirmed that the photodegradation of MB dyes was mainly contributed by the photogenerated electrons, as shown in the Additional File 1.

To further unveil the reaction kinetics of dye degradation and evaluate the involving reaction constant, three possible kinetic models, including first-order kinetic model, second-order kinetic model, and Langmuir–Hinshelwood kinetic model were examined, as given below,

First-order kinetic model [27]:

4 $$ {\mathrm{lnC}}_{\mathrm{t}}=\hbox{-} {\mathrm{k}}_1\mathrm{t}+{\mathrm{lnC}}_0 $$

Second-order kinetic model [28]:

5 $$ 1/{C}_{\mathrm{t}}={k}_2t+1/{C}_0 $$

Langumuir-Hinshelwood kinetic model [29]:

6 $$ \ln \left({C}_{\mathrm{t}}/{C}_0\right)+{K}_{\mathrm{L}}\left({C}_0\hbox{-} {C}_{\mathrm{t}}\right)=\hbox{-} {k}_3{K}_{\mathrm{L}}t $$

in which *k*_1_, *k*_2_, and *k*_3_ are the first-order, second-order, and Langmuir–Hinshelwood kinetic rate constants, respectively. Concentrations of dyes are denoted as *C*_0_ at reaction time = 0 and *C*_t_ at reaction time = *t*. In addition, *K*_L_ represents the constant of Langmuir absorption equilibrium. The results were summarized in Fig. 4c, where the corresponding correlation coefficients (R^2^) were 0.82 in first-order kinetic model, 0.96 in second-order kinetic model, and 0.71 in Langmuir–Hinshelwood kinetic model.

These findings clearly verified that the photodegradation kinetics of MB dyes using Cu_2_O-decorated SiNW arrays corresponded to second-order kinetic model. Thus, the explicit reaction constant could be evaluated, indicating that the most pronounced value appearing in the D-Cu_2_O/SiNW arrays, as demonstrated in Fig. 4d. These effects explained the significant influences on the spatial incorporation of Cu_2_O with SiNW hosts, which could act as a decisive rule on dye removal. Based on the detailed investigations, the photodegradation of MB dyes in the presence of Cu_2_O/SiNW arrays could be elucidated, as illustrated in Fig. 4e. The photoexcited electrons and holes from highly light-absorptive SiNWs could be efficiently separated, where the photogenerated electrons were energetically transferred to the conduction band of Cu_2_O nanoparticles, thus initiating the photodegradation of MB dyes. Therefore, the uniform Cu_2_O dispersions facilitated such carrier separation that enabled to effectively reduce the electron-hole recombination. Moreover, these dispersed features also allowed the high-penetration depth of incoming lights irradiated on nanowire structures, thus being favorable for the efficiency improvement in photocatalytic process. Finally, we have performed the repeated photodegradation experiments, termed as first run, second run and third run, and the corresponding XRD measurements along with corresponding SEM images after each photodegradation test were presented in the Additional File 1: Figure S5 and S6, respectively. The above results further evidenced that the photocatalysts could stably and reliably possess the photodegradation of MB dyes.

## Conclusions

In conclusion, we demonstrated an efficient visible-driven photocatalysts with a facile, inexpensive and reliable two-step electroless deposition for large-area production. These hybrid Cu_2_O/SiNW arrays with uniform Cu_2_O decorations were feasible for reducing the recombination of photogenerated carriers under visible-light irradiations. The investigations of photodegradation kinetics, along with facile synthetic process might benefit the development of high-performance and miniaturized photoactive substrates for diverse applications, including water treatment, water splitting, and other function devices.

## Additional file

Additional file 1: **Figure S1.** Adsorption diagram of A-Cu_2_O/SiNWs and D-Cu_2_O/SiNWs in the presence of MB dyes under the dark condition. Figure S2 High-magnification SEM image of D-Cu_2_O/SiNWs, which verified the successful coating of Cu_2_O nanoparticles on Si nanowires. Figure S3 Schematic interactions between incoming lights and various samples, including sole SiNWs, D-Cu_2_O/SiNWs and A-Cu_2_O/SiNWs, respectively. Figure S4 Radical-scavenging analysis of D-Cu_2_O/SiNW photocatalysts under various conditions. Figure S5 Degradation diagrams of repeated tests of D-Cu_2_O/SiNWs under the condition. Figure S6 XRD patterns along with the corresponding SEM images (DOCX 1015 kb)

## Abbreviations

A: Aggregated
D: Dispersed
DI: Deionized
EDS: Energy-dispersive X-ray
FESEM: Field emission scanning electron microscopy
SiNWs: Silicon nanowires
TEM: Transmission electron microscopy
XRD: X-ray diffraction
PL: Photoluminescent

## References

[1] Bessekhouad Y, Robert D, Weber JV (2005). Photocatalytic activity of Cu_2_O/TiO_2_, Bi_2_O_3_/TiO_2_ and ZnMn_2_O_4_/TiO_2_ heterojunctions. Catal Today.

[2] Lin S, Cui W, Li X, Sui H, Zhang Z (2017). Cu_2_O NPs/Bi_2_O_2_CO_3_ flower-like complex photocatalysts with enhanced visible light photocatalytic degradation of organic pollutants. Catal Today.

[3] Hu F, Zou Y, Wang L, Wen Y, Xiong Y (2016). Photostable Cu_2_O photoelectrodes fabricated by facile Zn-doping electrodeposition. Int J Hydrog Energy.

[4] Li H, Zhang X, MacFarlane DR (2015) Carbon quantum dots/Cu_2_O heterostructures for solar-light-driven conversion of CO_2_ to methanol. Adv Energy Mater 5:1401077

[5] Barreca D, Fornasiero P, Gasparotto A, Gombac V, Maccato C, Montini T, Tondello E (2009). The potential of supported Cu_2_O and CuO nanosystems in photocatalytic H_2_ production. ChemSusChem.

[6] Zhang J, Zhu H, Zheng S, Pan F, Wang T (2009). TiO_2_ film/Cu_2_O microgrid heterojunction with photocatalytic activity under solar light irradiation. ACS Appl Mater Interfaces.

[7] Chang Y, Teo JJ, Zeng HC (2005). Formation of colloidal CuO nanocrystallites and their spherical aggregation and reductive transformation to hollow Cu_2_O nanospheres. Langmuir.

[8] Huang Z, Fang H, Zhu J (2007). Fabrication of silicon nanowire arrays with controlled diameter, length, and density. Adv Mater.

[9] Sandu G, Kassa HG, Avram I, Gohy JF, Leclere P, Vlad A, Melinte S (2016). In Si-based three-dimensional assembly for lithium-ion batteries, E-MRS Spring Meeting.

[10] Puglisi Rosaria Anna, Lombardo Valentina, Caccamo Sebastiano (2017). Silicon Quasi‐One‐Dimensional Nanostructures for Photovoltaic Applications. Nanowires - New Insights.

[11] Peled A, Pevzner A, Soroka HP, Patolsky F (2014). Morphological and chemical stability of silicon nanostructures and their molecular overlayers under physiological conditions: towards long-term implantable nanoelectronic biosensors. J Nanobiotechnology.

[12] Yousong L, Junyi W (2012). Fabrication and photocatalytic properties of silicon nanowires by metal-assisted chemical etching: effect of H_2_O_2_ concentration. Nanoscale Res Lett.

[13] Yu L, Xiong L, Yu Y (2015). Cu_2_O homojunction solar cells: F-doped N-type thin film and highly improved efficiency. J Phys Chem C.

[14] Yousong L, Zhang X (2015). Preparation of Si/TiO2 heterojunction nanotube arrays via electrodeposition and their enhanced photocatalytic activity. Nanosci Nanotechnol Lett.

[15] Yousong L, Guangbin J (2014). Highly-active direct Z-scheme Si/TiO2 photocatalyst for boosted CO2 reduction into value-added methanol. RSC Adv.

[16] Li X, Bohn P (2000). Metal-assisted chemical etching in HF/H_2_O_2_ produces porous silicon. Appl Phys Lett.

[17] Chen CY, Wei TC, Lin CT, Li JY (2017). Enhancing formation rate of highly-oriented silicon nanowire arrays with the assistance of back substrates. Sci Rep.

[18] Hildreth OJ, Brown D, Wong CP (2011). 3D out-of-plane rotational etching with pinned catalysts in metal-assisted chemical etching of silicon. Adv Funct Mater.

[19] Li JY, Hung CH, Chen CY (2017). Hybrid black silicon solar cells textured with the interplay of copper-induced galvanic displacement. Sci Rep.

[20] Tsang CH, Li HH, Huang Z, To WK (2011). Fabrication of n-type mesoporous silicon nanowires by one-step etching. Nano Lett.

[21] Hildreth OJ, Lin W, Wong CP (2009). Effect of catalyst shape and etchant composition on etching direction in metal-assisted chemical etching of silicon to fabricate 3D nanostructures. ACS Nano.

[22] Rykaczewski K, Hildreth OJ, Kulkarni D, Henry MR, Kim SK, Wong CP, Tsukruk VV, Fedorov AG (2010). Maskless and resist-free rapid prototyping of three-dimensional structures through electron beam induced deposition (EBID) of carbon in combination with metal-assisted chemical etching (MaCE) of silicon. ACS Appl Mater Interfaces.

[23] Li S, Ma W, Zhou Y, Chen X, Xiao Y, Ma M, Wei F, Yang X (2014). Fabrication of p-type porous silicon nanowire with oxidized silicon substrate through one-step MACE. J Solid State Chem.

[24] Chen CY, Hsiao PH, Wei TC, Chen TC, Tang CH (2017). Well incorporation of carbon nanodots with silicon nanowire arrays featuring excellent photocatalytic performances. Phys Chem Chem Phys.

[25] Zhang X, Song J, Jiao J, Mei X (2010). Preparation and photocatalytic activity of cuprous oxides. Solid State Sci.

[26] Janczarek M, Kowalska E (2017). On the origin of enhanced photocatalytic activity of copper-modified titania in the oxidative reaction systems. Catalysts.

[27] Kim SH, Ngo HH, Shon H, Vigneswaran S (2008). Adsorption and photocatalysis kinetics of herbicide onto titanium oxide and powdered activated carbon. Sep Purif Technol.

[28] Tang CH, Chen KY, Chen CY (2018). Solution-processed ZnO/Si based heterostructures with enhanced photocatalytic performances. New J Chem.

[29] Kumar KV, Porkodi K, Rocha F (2008). Langmuir–Hinshelwood kinetics–a theoretical study. Catal Commun.

