# Supplementary material for: Efficient Photocatalysts Made by Uniform Decoration of Cu2O Nanoparticles on Si Nanowire Arrays with Low Visible Reflectivity
Source: Nanoscale Res Lett. 2018 Oct 4;13:312. doi: 10.1186/s11671-018-2735-7 (PMC6172162; doi:10.1186/s11671-018-2735-7)
Supplement: Supplementary file 1 — Figure S1. Adsorption diagram of A-Cu2O/SiNWs and D-Cu2O/SiNWs in the presence of MB dyes under the dark condition. Figure S2 High-magnification SEM image of D-Cu2O/SiNWs, which verified the successful coating of Cu2O nanoparticles on Si nanowires. Figure S3 Schematic interactions between incoming lights and various samples, including sole SiNWs, D-Cu2O/SiNWs and A-Cu2O/SiNWs, respectively. Figure S4 Radical-scavenging analysis of D-Cu2O/SiNW photocatalysts under various conditions. Figure S5 Degradation diagrams of repeated tests of D-Cu2O/SiNWs under the condition. Figure S6 XRD patterns along with the corresponding SEM images (DOCX 1015 kb) [file 11671_2018_2735_MOESM1_ESM.docx]

**Additional files**

**Efficient photocatalysts made by uniform decoration of Cu_2_O nanoparticles on Si nanowire arrays with low visible reflectivity**

**Chien-Hsin Tang^1^, Po-Hsuan Hsiao^1^ and Chia-Yun Chen^1,2,^***

^1^ Department of Materials Science and Engineering, National Cheng Kung University, Tainan 70101, Taiwan

^2^ Hierarchical Green-Energy Materials (Hi-GEM) Research Center, National Cheng Kung University, Tainan 70101, Taiwan


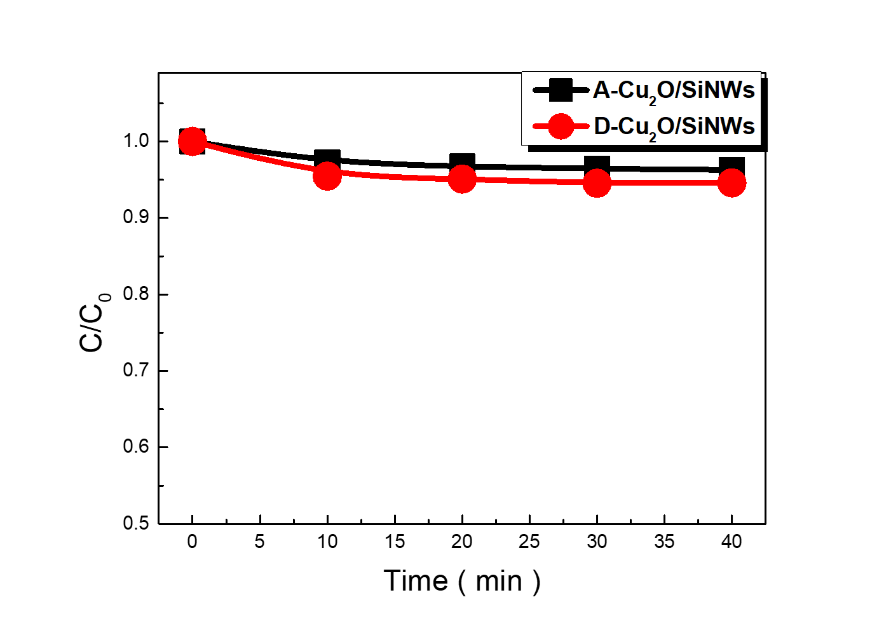


**Figure S1** Adsorption diagram of A-Cu_2_O/SiNWs and D-Cu_2_O/SiNWs in the presence of MB dyes under the dark condition. The results displayed the slightly superior adsorption ability of D-Cu_2_O/SiNWs in comparison with A-Cu_2_O/SiNWs.

**
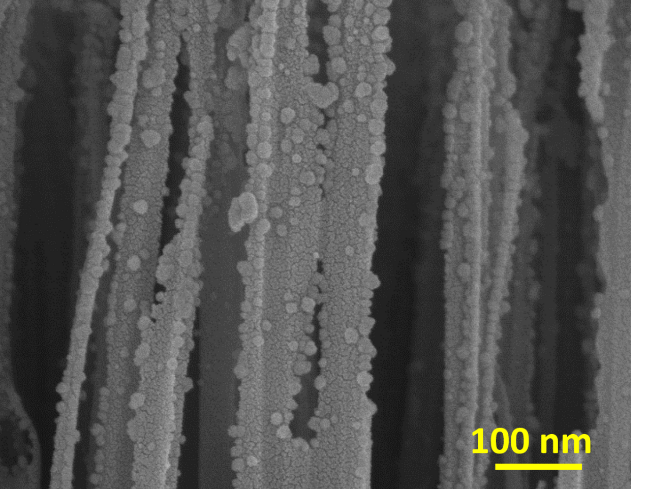
**

**Figure S2** High**-**magnification SEM image of D-Cu_2_O/SiNWs, which verified the successful coating of Cu_2_O nanoparticles on Si nanowires.


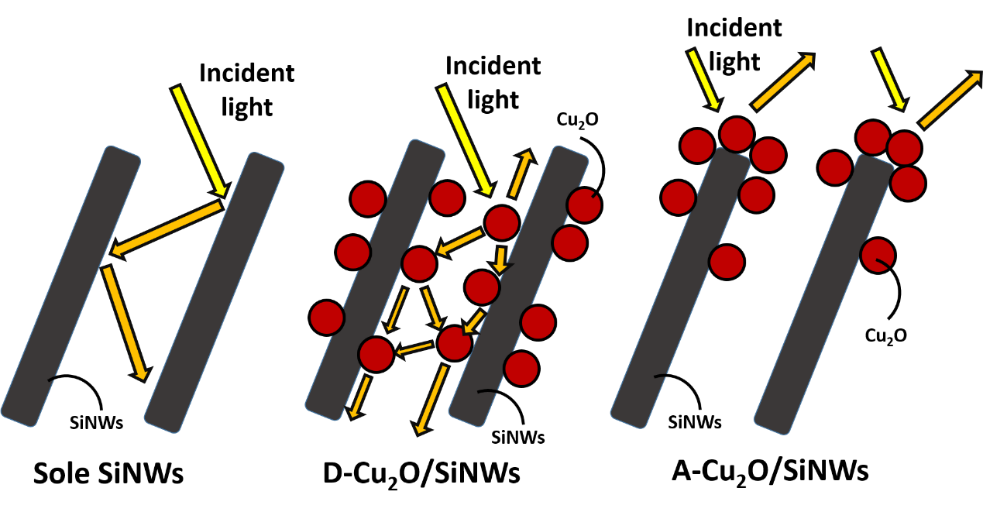


**Figure S3** Schematic interactions between incoming lights and various samples, including sole SiNWs, D-Cu_2_O/SiNWs and A-Cu_2_O/SiNWs, respectively.

**Figure S4** Radical-scavenging analysis of D-Cu_2_O/SiNW photocatalysts under various conditions: With no scavenger, EDTA addition, methanol addition and BZQ addition. The results clearly identified that the addition of EDTA could significantly affect the photocatalytic activity of photocatalysts, indicating that photogenerated electrons mainly initiated the photodegradation reactions of MB dyes.


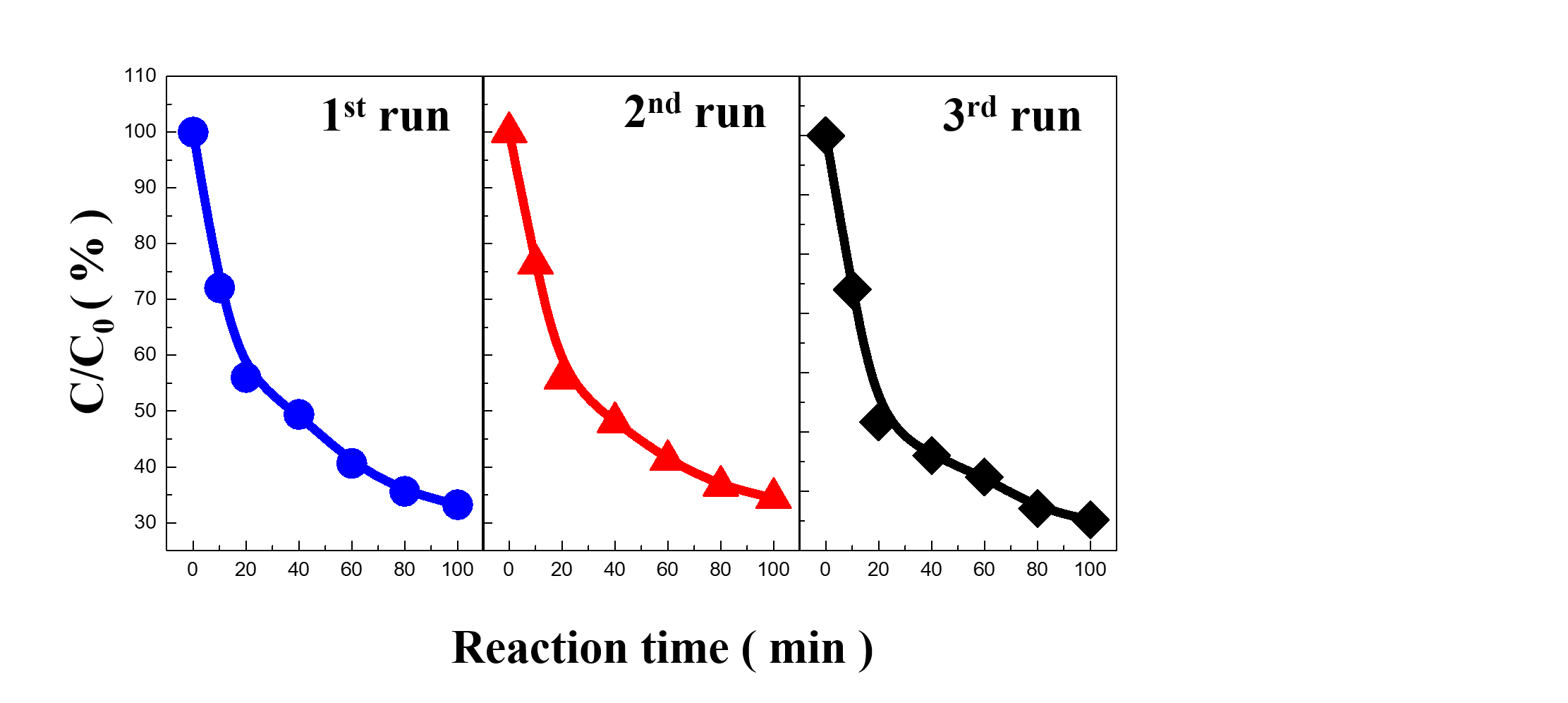


**Figure S5** Degradation diagrams of repeated tests of D-Cu_2_O/SiNWs under the condition: 1^st^ run photodegradation, 2^nd^ run photodegradation and 3^rd^ run photodegradation.

**
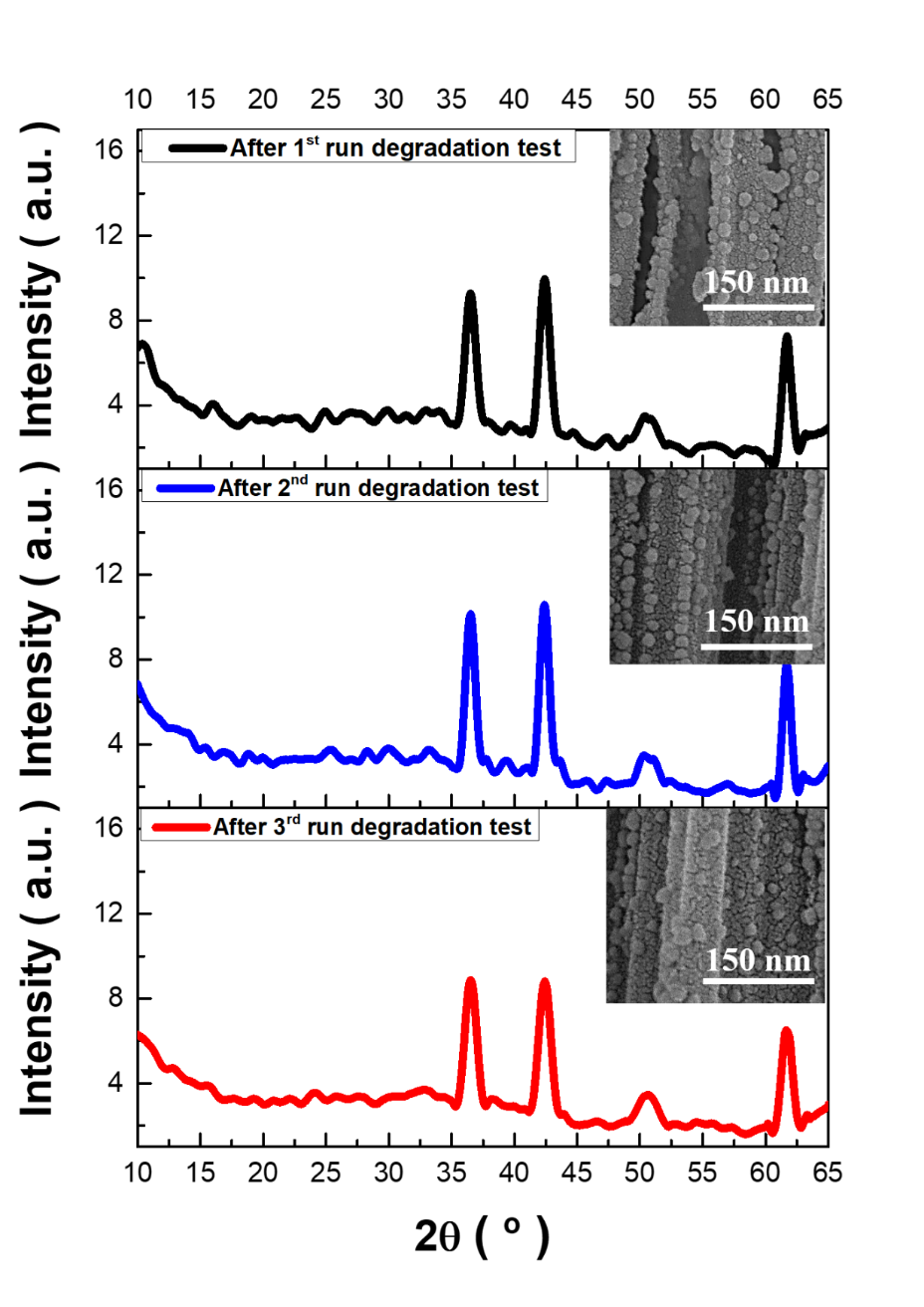
**

**Figure S6** XRD patterns along with the corresponding SEM images of D-Cu_2_O/SiNWs after the 1^st^ run photodegradation, 2^nd^ run photodegradation and 3^rd^ run photodegradation experiments. The results evidenced the unchanged XRD patterns and morphologies of used photocatalysts.
